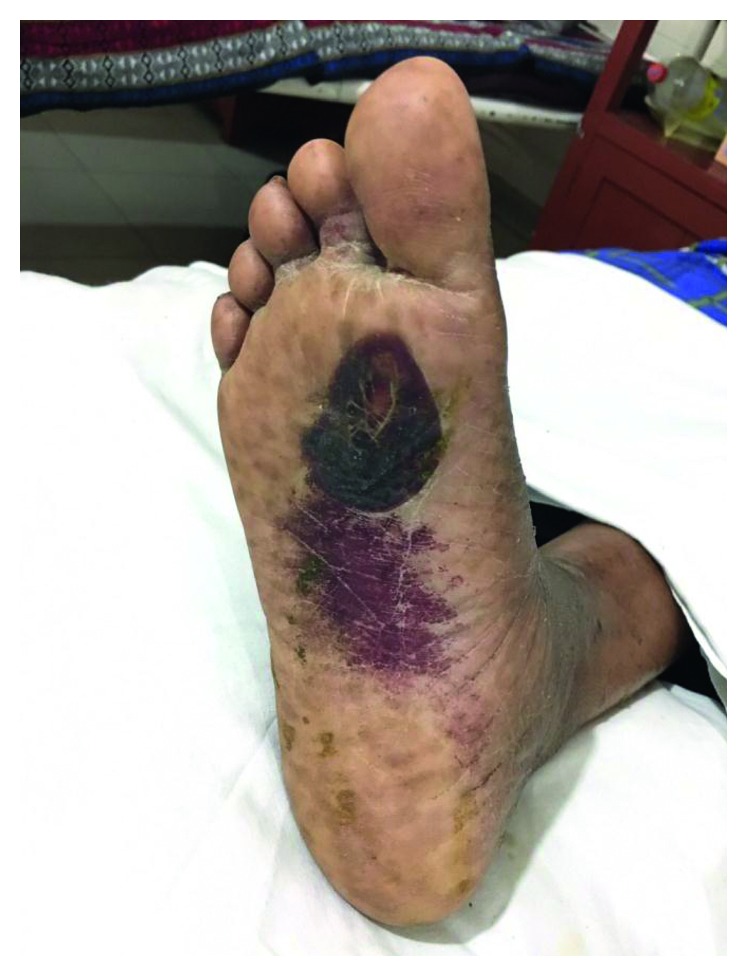# Cardiogenic Shock: An Unusual Initial Presentation of Churg-Strauss Syndrome

**DOI:** 10.1155/2018/2076837

**Published:** 2018-04-01

**Authors:** M. Apirami, J. A. Pratheepan, T. Kumanan, M. Guruparan, G. Selvaratnam

**Affiliations:** ^1^Teaching Hospital Jaffna, Jaffna, Sri Lanka; ^2^Teaching Hospital Jaffna, Faculty of Medicine, University of Jaffna, Jaffna, Sri Lanka

## Abstract

Churg-Strauss syndrome (CSS) is a rare autoimmune condition, characterized by necrotizing extravascular eosinophil rich granulomatous inflammation of the tissues and disseminated small-medium sized vessel vasculitis in a patient with bronchial asthma and tissue eosinophilia. Though pulmonary involvement is the predominant feature of CSS, extra pulmonary involvement, in particular, cardiac involvement, denotes an adverse outcome. Here we report a 50-year-old female who presented with cardiogenic shock due to an acute coronary event as the initial manifestation of CSS. A subsequent coronary angiogram revealed normal epicardial coronaries. She was a patient with bronchial asthma and developed vasculitic rash, bilateral sensory motor polyneuropathy, and migratory peripheral lung field shadows in the background of peripheral eosinophilia during the course of the illness. She was diagnosed as having CSS based on ACR criteria and aggressively treated with immunosuppressants according to her Five-Factor Score and has shown prompt response to therapy. This case report adds to the literature another rare initial presentation of CSS to the existing array of its clinical manifestations.

## 1. Introduction

Churg-Strauss syndrome (CSS) is a rare multisystem disorder characterized by necrotizing vasculitis and extravascular granuloma formation. The characteristic presentation is bronchial asthma with hypereosinophilia.

CSS classically affects the arteries of pulmonary vasculature and skin. Involvement of extra pulmonary component is often not prominent as an initial presentation but often is the key determinant of prognosis in a patient with CSS.

One of the rare extra pulmonary manifestations is coronary involvement which contributes to adverse prognosis and causes significant mortality. Approximately 50% of deaths among untreated CSS patients are due to cardiac involvement [1].

Here, we report a woman with severe coronary artery spasm complicated by acute coronary syndrome and cardiogenic shock as an initial presentation of CSS.

## 2. Case Presentation

A 50-year-old female, a known patient with moderate persistent bronchial asthma for five years, had frequent exacerbations of the illness for a year while on regular medications.

She presented to the medical emergency with sudden onset of breathing difficulty associated with chest tightness of 30 minutes duration. On admission, she had a blood pressure of 80/50 mmHg and a regular pulse rate of 122/min. Chest examination revealed polyphonic rhonchi and scattered bilateral crepitations.

A bedside 12-lead ECG on admission showed ST segment elevations in V1–V3. Cardiac troponin was elevated (4.9 IU/L), and a 2D echocardiogram revealed anterior and septal wall hypokinesia with an ejection fraction of 30%.

Though there were no known risk factors for coronary artery disease, she was diagnosed and managed as having acute coronary syndrome with cardiogenic shock. While in hospital, troponin levels fluctuated, suggesting recurrent coronary events. After two weeks of ventilator support and ICU care, she was discharged with regular medications.

The coronary angiogram performed two weeks after discharge revealed normal epicardial coronary vessels.

**Figure 1:**
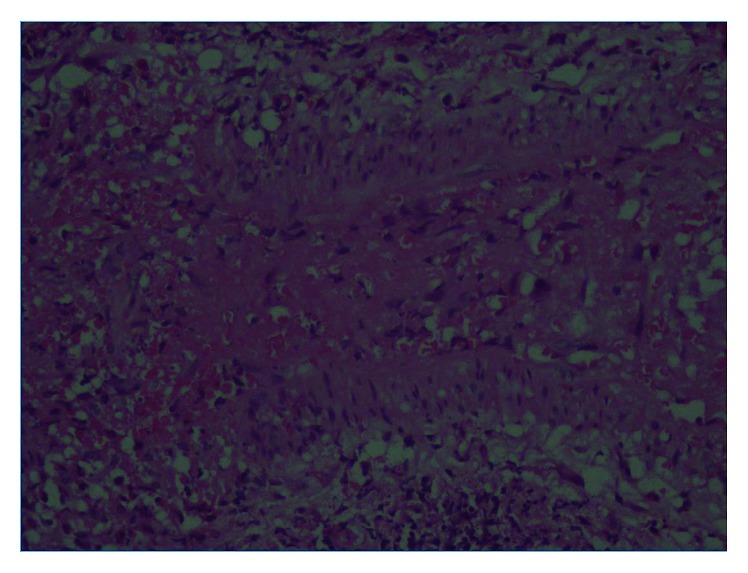


Two months after the initial presentation, she developed progressive bilateral sensory motor peripheral neuropathy. Sural nerve biopsy (Figure 1), performed subsequently showed a vasculitic peripheral neuropathy with high eosinophil infiltrate. On general examination, she was also found to have a vasculitic rash over her lower extremity (Figure 2).

The FBC showed 17% eosinophils with the absolute count of 1900 cells. She had transient migratory consolidations in peripheral lung fields on her CXR which was poorly responding to antibiotic treatment. HRCT chest revealed a right-sided basal opacity with bronchiectatic changes.

Her ESR was 64 and CRP was23. Serology screening for autoantibody including ANCA antibodies was negative. She never had any active sediment in the urine to suggest renal involvement.

Based on the ACR criteria, she was diagnosed as having CSS and the Five-Factor Score was >2. She was initiated on parenteral methylprednisolone 500 mg for three days and then followed by oral prednisolone of 45 mg/day and concurrently cyclophosphamide pulse therapy was also commenced.

With immunosuppression, her asthma got better, her skin lesion regressed, eosinophils normalized, and peripheral neuropathy improved.

At present, she is symptom free. Azathioprine is to be added as a steroid sparing agent for maintenance therapy.

## 3. Discussion

CSS is a rare multisystem disorder characterized by small to medium vessel vasculitis in a patient with asthma and tissue eosinophilia. The incidence is about 0.5 to 6.8 per million, and the prevalence is estimated to be around 10.7 to 14 per million [2].

Though the exact pathogenesis of CSS is poorly understood, CSS patients are found to have high prevalence of HLADRB1, HLADRB4, and several abnormalities in their immune functions as well.

40–60% of CSS patients are positive to ANCA in particular the p-ANCA. But the exact role of ANCA in the pathogenesis of CSS is not known. Other noted immune abnormalities are heightened TH1 and TH2 activity, abnormal eosinophil response, and reduced production of IL10. Some of the CSS patients have hypergammaglobulinaemia and positive rheumatoid factor as well, which indicate the role of humoral immunity in the disease process.

CSS typically manifests in three phases, but they are often indistinguishable. The prodromal phase is the phase where the patients usually present with asthma, allergic rhinitis, and sinusitis, in their second to third decade of life. The eosinophilic phase is the phase in which patients will have high blood eosinophilic count and eosinophilic infiltration of multiple organs. Finally, the vasculitic phase manifests with nonspecific constitutional symptoms and features of multiple organ involvement.

One of the rare extra pulmonary involvements is the cardiac involvement which accounts for 17–60% among CSS patients. These patients show high eosinophilic count and proportional disease activity [3].

CSS patients with negative ANCA activity are found to have increased incidence of cardiac involvement in contrast to the ANCA positive patients [4]. Hence, it is reasonable to assume that ANCA's role is minimal and probably activated eosinophils play a major role in the pathogenesis of cardiac damage. Activated eosinophils releases toxic granules which secrete eosinophil babic protein and eosinophilic catarophic proteins: these proteins cause tissue damage [5]. Cardiac manifestations can be the result of acute myocardial damage or intermediate thrombus and late fibrosis formation or valvular damage [5].

Cardiac complications may vary from subtle with minimal clinical significance to an extent to cause life threatening events as happened in this patient [6]. Potentially serious cardiac complications include endocarditis, myocarditis, acute pericarditis, coronary vasculitis, myocardial ischemia, conductance disturbance, arrhythmia, valvular dysfunction, eosinophilic pericardial effusion, and sudden death. They may even progress to left ventricular dysfunction, constrictive pericarditis, and myocardial fibrosis as a late sequelae [5, 6].

Among the above manifestations, coronary artery spasm in a patient with CSS is an extremely rare initial manifestation. Hyper contractility of the vascular smooth muscle in the background of high eosinophils is the main cause of coronary spasm [7]. But exact pathogenesis is unclear. Vasodilators are the mainstay of treatment in usual vasospasm, but in patients with CSS induced vasospasm, vasodilators have little role in the treatment [8]. These patients will respond to immune suppresser therapy better.

Coronary spasm was the first manifestation in this patient that manifested as cardiogenic shock due to an acute coronary syndrome in a background of normal epicardial coronary vessels. The 2D echocardiogram after the coronary event showed anterior and septal wall hypokinesia and severe LV dysfunction. Even while on vasodilators, she developed recurrent episodes of coronary events, which settled after initiation of immunosuppressive therapy.

The diagnosis of CSS was made according to ACR criteria. Based on the Five-Factor Score, she was initiated with aggressive treatment (her Five-Factor Score was >2).

She responded promptly to the treatment. After a period of 5 months of treatment, the disease is in remission and the LV function had significantly improved with an LVEF of 60%.

## Figures and Tables

**Figure 2:**